# Towards Responsible Rebellion: How Founders Deal with Challenges in Establishing and Governing Innovative Living Arrangements for Older People

**DOI:** 10.3390/ijerph17176235

**Published:** 2020-08-27

**Authors:** Katja M. Rusinovic, Marianne E. van Bochove, Suzanna Koops-Boelaars, Zsuzsu K.C.T. Tavy, Joost van Hoof

**Affiliations:** 1 Urban Social Development, Faculty of Public Management, Law & Safety, The Hague University of Applied Sciences, Johanna Westerdijkplein 75, 2521 EN Den Haag, The Netherlands; 2 Department of Health Care Governance, Erasmus School of Health Policy & Management, Erasmus University Rotterdam, Burgemeester Oudlaan 50, 3000 DR Rotterdam, The Netherlands; vanbochove@eshpm.eur.nl (M.E.v.B.); koops@eshpm.eur.nl (S.K.-B.); 3 Faculty of Health, Nutrition & Sport, The Hague University of Applied Sciences, Johanna Westerdijkplein 75, 2521 EN Den Haag, The Netherlands; z.k.c.t.tavy@hhs.nl; 4 Faculty of Social Work & Education, The Hague University of Applied Sciences, Johanna Westerdijkplein 75, 2521 EN Den Haag, The Netherlands; j.vanhoof@hhs.nl; 5 Institute of Spatial Management, Faculty of Environmental Engineering and Geodesy, Wrocław University of Environmental and Life Sciences, ul. Grunwaldzka 55, 50-357 Wrocław, Poland

**Keywords:** housing, older people, elderly, assisted living facilities, seniors, homes, group living, dwellings, regulations, rebellion, governance, law

## Abstract

In the Netherlands, there is an increasing need for collective forms of housing for older people. Such housing bridges the gap between the extremes of living in an institutionalised setting and remaining in their own house. The demand is related to the closure of many residential care homes and the need for social engagement with other residents. This study focuses on housing initiatives that offer innovative and alternative forms of independent living, which deviate from mainstream housing arrangements. It draws on recent literature on healthcare ‘rebels’ and further develops the concept of ‘rebellion’ in the context of housing. The main research question is how founders dealt with challenges of establishing and governing ‘rebellious’ innovative living arrangements for older people in the highly regulated context of housing and care in the Netherlands. Qualitative in-depth interviews with 17 founders (social entrepreneurs, directors and supervisory board members) were conducted. Founders encountered various obstacles that are often related to governmental and sectoral rules and regulations. Their stories demonstrate the opportunities and constraints of innovative entrepreneurship at the intersection of housing and care. The study concludes with the notion of ‘responsible rebellion’ and practical lessons about dealing with rules and regulations and creating supportive contexts.

## 1. Introduction

All over the world, people live longer and are generally in better health than previous generations of their age. Age-friendly cities and communities can help improve the quality of life of older citizens. An age-friendly city or community is a place where older people are actively involved, valued, and supported with infrastructure and services that effectively accommodate their needs [1,2,3]. Housing is one of the key domains of establishing age-friendly cities. Creating appropriate housing for older people is one of the major challenges facing Western countries [2,4,5]. Ever-increasing numbers of older people live independently and remain part of the society at large, also referred to as ageing-in-place. This trend is not just related to the preferences or wishes of older people themselves. Government measures, such as reforms in long-term care, also play an important role [6]. When taking a closer look at the Netherlands, there are several challenges related to housing for older people and the housing career of this cohort [7]. In recent years, for instance, many residential care homes, which bridged the gap between one’s own home and a nursing home, have been closed. Some of these vacant premises have been innovatively converted into facilities for independent living [8]. Given the changes in policies, a significant group of older people in need for collectivity are in danger of getting left out. In addition, there are many older people who do not need continuous care and support, but who are nevertheless seeking the safety and jointness of a collective form of housing (sometimes referred to as senior co-housing communities) [9]. A collective form of housing is a type of residence inside a larger building that has housing as its main function. It consists of several residential units, and a minimum of two households voluntarily share at least one living space, and each have at least one private living space. Residents of co-housing communities live in their own apartments but undertake activities together and support one another. There is an administrative separation between the need for housing on the one hand, and the need for care on the other. This is reflected in a lease contract for the home and a separate contract for the provision of care, only if needed by the older tenant. Some of these types of age-friendly housing bridge the gap between the need for ageing-in-place and living in institutional care facilities, as was the case with the former residential care facilities [7].

The Dutch government expects that municipalities, social housing associations and market parties take more action in the coming years, and that more innovative forms of housing that bridge the gap between ageing-in-place and institutional care facilities are developed [10], p. 40. In particular, the current supply of intermediate forms of housing for older people with low and middle incomes is limited in terms of capacity and insufficiently innovative [10], p. 39. However, this does not mean that innovative in-between solutions for lower and middle-income groups are absent. In recent years, various promising initiatives have been launched by directors of established social housing associations and by social entrepreneurs. A social enterprise delivers a product or service just like any other enterprise and has a revenue model, but earning money is a means of achieving a societal mission. For instance, in the case of a social housing association, revenues are re-invested in the quality, affordability and availability of social housing. Founders of innovative initiatives, both directors and social entrepreneurs, often present themselves in catchy one-liners as contrary pioneers, who go against the prevailing views and practices [11]. They emphasise the differences between their own initiatives, presented as innovative and positioned outside traditional frameworks, and the procedures and products of other parties, which they depict as old-fashioned and rigid. When establishing innovative collective housing concepts, directors of social housing associations and social entrepreneurs are faced with a multitude of challenges, such as the large number of national directives which need to be adhered to, and the involvement of numerous stakeholders in concepting and decision making [12]. How founders deal with challenges of establishing and governing innovative new living arrangements is a new field of study. Van Straaten et al. [13] studied the barriers, lessons learnt and pathways towards solutions among founders of new housing arrangements for older people in the Netherlands. The founders experienced a variety of challenges in working together with municipalities, as every municipality has its own methods and procedures. Founders with a track record find it easier to work with municipalities. This can be explained by their experience with dealing with all sorts of organisations as well as their rules and regulations.

In studying how founders deal with internal and external regulations, one can draw on the literature from the healthcare domain, where ‘positive deviance’, ‘tempered radicals’ and ‘rebellion’ have recently received attention [14,15,16,17]. In this study, we use the concept of ‘rebellion’ to refer to those actors who identify with and are committed to their organisations and possess the reflexivity and innovativeness to act otherwise and to find different institutional solutions to do better. This definition is based on central elements of both the concepts of ‘tempered radicals’ as well as ‘positive deviants’ [16,17]. The existing literature focused on individuals or teams within traditional care organisations, such as hospitals and nursing homes, who dare to do things differently with the aim of providing a better quality of care. Although this article studies rebellion in a different context, three findings from the existing literature are particularly relevant.

First, *rebels use and create space for doing things differently*. In the Dutch public and political debate, it is often argued that governmental rules and regulations cause the regulatory pressure that many healthcare professionals experience. However, research conducted in Dutch nursing homes showed that—although some of the rules, which were experienced as unnecessary, were indeed formulated by external parties such as ministries or inspectorates—many rules were actually formulated by the care organisations themselves [15]. Instead of complaining about such rules, or blindly obeying them, ‘rebellious’ directors, middle managers and care professionals working in the nursing homes tried to trace the origin and function of burdensome rules and to bend, change or work around them. Following this strategy enabled some (subunits of) organisations to increase the quality of care by doing things differently in a reflexive, well-considered way.

Second, *rebels shake up organisations, but do not intend to harm or leave them*. Rebels are able to bring about meaningful change by bending or ignoring certain rules [18]. The actions of these rebels can be referred to as ‘positive deviance’ in the sense that their behaviour deviates from the norm in such a manner that it effectively addresses certain problems [16]. However, this does not mean that they oppose rules in general. Wallenburg and colleagues, who focused on rebels in hospital settings, argue that ‘positive deviants (…) tend to shake up the organization, but do not intend to harm or leave the organisation, rather seeking to care for the organization and its purposes’ [14], p. 871. In other words, these rebels change the system from within, rather than as complete outsiders.

Third, *rebellion is not only a characteristic of individuals but also of networks*. Related to the previous point, the literature shows that rebels need allies. While rebellion is sometimes studied on an individual level [17], recent studies showed that rebellious behaviour is often displayed in teams [14,15]. Team members discuss which rules are vital to obey, which ones can be changed, and which ones can be ignored. Rebellious individuals, thus, need rebellious teams, which not only include ‘deviants’ but also ‘guardians’. Such guardians ensure the continuity of the operation, reduce the chance for potential risk or damage to the organisation and avoid potential disasters [19]. Rebellious teams, in turn, need organisational contexts that enable them to achieve their goals [14].

The above-discussed findings are relevant for the present study, which aims to research how founders deal with challenges of establishing and governing innovative new living arrangements in the highly regulated context of the Netherlands. In studying how founders deal with regulatory challenges in the establishment and governance of their initiatives, attention will be paid to how founders perceive rules, and what founders do when they come across burdensome rules that do not seem to be related to good quality services. Moreover, it is investigated how the founders relate to other relevant actors, such as various stakeholders within their initiatives, partner organisations and external regulatory bodies. Although this study builds on the findings of earlier studies, it differs from the existing literature on rebellion and innovative housing arrangements in three important ways. While earlier studies focused on rebellious individuals or teams within ‘traditional’ organisations, this study deals with the initiatives of social entrepreneurs who have established ‘rebellious’ organisations. Building networks and finding allies might be even more important—and more difficult—for them than for actors who work in established organisations. A second difference from earlier studies on rebellion is that this study includes initiatives on the cutting edge of housing and care, which likely makes dealing with regulatory pressure and knowing how to create room to manoeuvre even more important. Third, this study adds to earlier studies on ‘innovative models for ageing-in-place’ [20], which mainly focused on the characteristics of various types of arrangements [21]. This study does not aim to generate a typology of new living arrangements, but rather to provide insight into the perceptions and practices of the founders in establishing and governing such arrangements. By combining a focus on rebellion and innovative living arrangements, this study contributes to both fields of the literature.

In the following sections, the selection of ‘rebellious’ initiatives and important aspects of our qualitative research design are outlined. Thereafter, research findings are presented. In the discussion, the term ‘responsible rebellion’ is introduced. This is followed by a reflection on the implications of ‘responsible rebellion’ for theory and practice.

## 2. Methodology

In order to obtain a closer understanding of the challenges experienced by founders in setting up group living arrangements for older people, a qualitative study was conducted. Data acquisition took place through qualitative (face-to-face) interviews. The decision-making processes in innovative collective housing projects were examined based on the experiences of the founders involved and (un-)successful cases. In addition, the challenges that actors have to deal with regarding (1) internal and external regulations, and (2) establishing (in)formal partnerships and (3) how this is governed by the directors and the supervisory board, were identified.

### 2.1. Participants, Settings and Interviewing

A total of 17 key figures from the domain of housing for older people were interviewed, among which were social entrepreneurs, directors and members of the supervisory board (Table 1). These founders were recruited through the personal and professional networks of our consortium partners, including small and medium-sized enterprises, knowledge institutes, and stakeholder associations (VTW (Vereniging van Toezichthouders in Woningcorporaties. English: Association of Supervisors of Social Housing Associations), NVTZ (Nederlandse Vereniging van Toezichthouders in Zorg en Welzijn. English: Dutch Association of Supervisors in Care and Welfare) and Aedes (Aedes—Vereniging van Woningcorporaties. English: Aedes—Dutch Association of Social Housing Organisations. Together, Aedes members manage 2.4 million dwellings, constituting 32% of the total housing stock in the Netherlands)), which all have an extensive network in the field of senior co-housing. Purposeful sampling was applied as a technique in selecting the cases. This technique is widely used in qualitative research for the identification and selection of information-rich cases [22]. It involves identifying and selecting participants who are knowledgeable about or experienced with a phenomenon of interest [22]. Participants were selected because they were mentioned by our consortium partners or other participants as someone known as ‘rebellious’, as someone who works differently, or who possesses the reflexivity and innovativeness to act otherwise.

The initiatives of the founders are all examples of living arrangements positioned on the housing continuum in-between ageing-in-place and institutionalised housing and built for low and middle-income households. In a few cases, the living arrangement is aimed at a specific group of older people, such as people with dementia or older people of ethnic minority groups. Three examples are provided to show the diversity of initiatives included, and to illustrate that the innovation of such housing concepts lies in an innovative approach to both the architectural aspects and the organisation of—and interaction with—care and welfare services.

In one of the housing initiatives older people and students live together. Here, a large social housing association transformed the existing real estate—a former residential care home—into an intergenerational living community. This happened in close collaboration with the local community. The social housing association formed a partnership with a local care partner that provides care for the residents if needed. The housing association leads similar innovative transformation processes in other municipalities in the Netherlands. 

A second example is a social enterprise that establishes small living communities for more or less self-reliant middle-income seniors. Founded in 2012, the concept originally consisted of a courtyard community, in which multiple smaller housing units make use of an inner courtyard. Here, according to the concept, older people can live independently and age-in-place, as people can help and accompany each other if wanted or needed. The social enterprise works with collective private commissioning, which means a group of private investors, or future residents, is formed in order to finance and further shape the development of the housing facility. Various similar communities were established throughout the country. 

A third example is a social enterprise that offers short-term stay in guesthouses where informal care is provided. Older people, who have undergone treatment in a hospital or rehabilitation centre and no longer receive a formal health assessment entitling them to publicly funded ‘care with residence’ can stay in a guesthouse located in their own neighbourhood. Whenever they need formal care, their familiar professional carers stop by.

There is a large diversity in the sample of participants. The majority (n = 9) of the participants is a director, followed by social entrepreneurs (n = 6). Two members of a supervisory board of a living arrangement were interviewed as well, as these supervisors were involved in the planning and supervision of innovative housing projects. The research strategy to include participants with different positions in the sample, added richness to the data, as it gives different stakeholder perspectives. The sample included 13 males and 4 females. Of the 17 participants, three were members of ethnic minority groups, but for reasons of privacy and potential identification, these data are not shown in Table 1. The large number of participants were aged between 50 and 60 years old (n = 7). Only three were younger than 50 years of age. 

The interviews took place at the offices of the participants or at an agreed-on convenient venue between July 2019 and January 2020. All interviews were conducted face-to-face, recorded and transcribed verbatim. The interviews lasted for approximately one hour. 

### 2.2. Topic Lists

Interviews were conducted based on a topic list. The themes of the topic lists were derived from a literature study, including the work by Meyerson [17] and Wallenburg et al. [14] and included dealing with rules and legislation, forming partnerships and networks, and the term rebellion. The topic list was discussed with a number of consortium partners, who are experienced in the field of governance of (collective) housing and adjusted accordingly. The topic list was divided into a structured and a semi-structured section. There were three topics lists, one for social entrepreneurs, one for directors, and one for members of a supervisory board, based on different roles that these positions bring (Supplementary Materials). First, background information, including questions on date of birth, and level of education was collected in order to gain insight into variation within the study population. The topic list contained topics grouped around a number of main themes: (i) the collective living arrangement, (ii) the concept of rebellion, (iii) dealing with rules and regulation and (iv) accountability. The topic list allowed us to gain insight into the participants’ experiences, motives, actions and (in)formal partnerships. The focus during the interviews differed, depending on the participants’ position, knowledge and expertise.

### 2.3. Data Analysis

The interviews were anonymised, elaborated and thematically analysed. For the thematic analysis, a qualitative analysis software package (Atlas.ti) was used.

In line with the ‘abductive analysis’ approach developed by Tavory and Timmermans [23], the analysis consisted of an iterative process of working with the empirical materials in relationship with the literature on rebellion and innovative living arrangements. This approach includes both deductive and inductive reasoning. Based on the existing literature, codes were used, such as ‘rules’, ‘regulations’ and ‘context’, but some codes were generated inductively (for instance, the different ideas respondents had about the term ‘rebellion’). During the coding process, three central themes emerged: four central elements of what it means to act ‘rebellious’; dealing with the institutional context, consisting of rules and regulations; and creating supportive (external and internal) contexts. 

The quotations were translated into English by the authors for the purpose of this article. Contextual information (including names of Dutch public bodies) has been simplified for a better understandability by an international audience.

### 2.4. Ethics

Since the data and selected cases contain many sensitivities, maintaining participant confidentiality and privacy are of the utmost importance. Confidentiality was addressed during the data collection, data cleaning and dissemination of the research results [24]. This confidentiality is also reflected in the limited case description of the chosen living arrangements. Prior to the phase of data collection, informed consent was obtained from the participants. Participants were asked to read and sign the informed consent statement at the beginning of the interview. After the data were collected, a ‘clean’ data set was created, one which does not contain information that identifies the participants, such as names or address details. The participants’ and organisations’ names were replaced with pseudonyms. Finally, in presenting the findings by using specific quotations and examples, it was explicitly considered if this could lead to the identification of a participant via deductive disclosure. If so, we removed additional details in the quotations. The quotations included in this article can only be traced by the researchers, based on the participant code.

## 3. Results

The following themes are described in the sections below: (1) Four central elements of rebellion: keeping the higher goal in mind; taking action; learning by trial-and-error; and thinking critically. (2) Institutional context: dealing with rules and regulations. (3) Creating supportive external and internal contexts.

### 3.1. Rebellion: Four Central Elements

The founders were asked what they thought of the term rebellion and whether or not they identified themselves or their initiatives as being rebellious. About half of the participants were positive about the term. Some of them said that they identified themselves as a rebel, and/or their initiatives as rebellious, while others said that they found it difficult to label themselves as rebels. According to them, that is for others to judge. About a third of the participants had negative associations towards the term rebel. For instance, they related it to warfare or earning success at the expense of others. The remainder of the participants had neither positive nor negative thoughts about the term. Whether or not they liked the term, various participants said that they preferred other terms, such as ‘radical’ (in the sense of breaking with the past), ‘entrepreneur’ (who takes risks) and ‘pacesetter’ (who involves other people and tries to enthuse them).

Despite their different opinions about the terms rebel and rebellious, all participants said that they and their initiatives dare to do things differently. In their explanations of their innovative approach, four central elements could be identified: keeping the higher goal in mind; taking action; learning by trial-and-error; and thinking critically instead of blindly following rules.

The founders talked passionately about the reasons for starting their initiatives and the societal values they pursued. Various founders shared personal stories about their parents who were the reason for starting the initiative: when their parents became older and in need of care, the founders were not satisfied with traditional care services and decided to create an alternative. One founder (Participant 3) said that, in setting-up his initiative, he always kept his mother in the back of his mind. Another founder (Participant 2) said that ‘*providing older people a safe and sociable home*’ is the main objective and he tries to go ‘*as far as possible*’ in achieving that. Others, for instance, describe the quality (Participant 8) and the continuity (Participant 4) of care as the main objectives. According to the participants, rebellion is a means to an end and should never become a goal in itself.

*“I would not describe it as kicking against something, because that has a negative association; the kicking becomes a goal in itself. For me, it is continuously thinking about what I am doing and trying to do it in different ways.”* (Participant 6)

*“It should not be about the rebellion itself. That you want to be in the spotlights for a moment. It should really be about a specific need or question. Something you want to find a solution for.”* (Participant 1)

The personal stories of various founders showed that they were frustrated and sometimes even angry about how housing for older people was organised in the Netherlands. What makes them stand out from many other people who expressed their opinion, was that they decided to take action to change the status quo. Taking action, or in the words of various participants, ‘just start doing it’, is a central element of rebellion. Rather than endless thinking, talking and planning, they tried to put their idea into practice right away.

*“I would define rebellion as taking initiative or setting up something and continue doing that, even if it doesn’t fit existing frameworks. That is rebellion for me: We are just going to do it. The developer says it doesn’t fit, or the municipality says it doesn’t fit the way they financially organised care. Despite all this, you say: ‘We are just going to do it. The idea is good, so we are doing it’.”* (Participant 1)

*“What makes it rebellious is that we said: ‘We are just going to do it. We will see how it turns out’.”* (Participant 5)

According to various founders, an action-directed mentality implies learning by doing. Because their initiatives were innovative and broke with existing traditions, there was no existing pathway founders could—or wanted to—follow. By experimentation and trial-and-error, they found out what worked and what did not.

*“It is about searching how you can do things differently. You try different things and sometimes you don’t succeed.”* (Participant 6)

Learning by trial-and-error asks for certain personal and organisational characteristics, such as being persistent and daring to take risks.

*“An entrepreneur sometimes takes risks, that is part of being an entrepreneur. When you say: ‘I believe in it’, then we believe in it and we go for it and we also take risks. That is also something that I will always do.”* (Participant 7)

One final often-mentioned element of rebellion was thinking critically instead of blindly following existing rules or traditions.

*“When organisations literally do what is prescribed by the rules, I find them so unimaginative.”* (Participant 9)

*“I always call it ‘healthy rebellion’. You need people who try colour outside the lines and who see what happens next.”* (Participant 13)

The participant quoted above called the critical thinking ‘healthy rebellion’, another participant said his behaviour was ‘friendly rebellious’ towards more traditional stakeholders in the field of housing and care (Participant 16). With these specifications, founders tried to make it clear that they did not want to rebel in an aggressive or irresponsible way. In the next section, their critical but responsible stance towards existing rules and regulations is discussed in further detail.

### 3.2. Institutional Context: Dealing with Rules and Regulations

Although founders mentioned that it is important to ‘just start’ and take the initiative, they experienced all sorts of limitations and barriers, especially with regard to the institutional context. Founders indicated that the amount of legislation and regulations can be an important barrier in setting up new housing initiatives. The realisation of such new housing initiatives is often complex. Many times, local regulations and procedures are simply not attuned to initiatives that deviate from the ‘ordinary’. One of the participants, who was in the process of setting up a new housing initiative, shared the following:

*“Municipalities are very bureaucratic organisations. I need to fill out an application form, and thereafter, someone from the municipality has to agree on it, and then someone else needs to find a budget for it. These steps take a lot of time.”* (Participant 1)

Founders also ran into the organisation and internal procedures of municipalities. Housing initiatives for older people are often seen within the indivisible scope of ‘housing and care’, such as in the case of nursing homes in which both the provision of shelter and care services are taken care of by a single organisation. At the municipal level—especially in larger municipalities—housing and care are usually two different policy fields, whereby cooperation and coordination between these departments is not always self-evident. As a result, decisions are made slowly because the decision-making process has to flow through the right channels:

*“Each department has its own set of rules […] Everything is formally written down. The civil servant in question is certainly not at risk. No one dares to take any risks. One is afraid to operate beyond the existing frameworks.”* (Participant 17)

Another barrier experienced by founders, related to the procedures mentioned above, is that the number of rules and regulations result in a rigid institutional system with few opportunities to conduct experiments:

*“The main problem is that due to all the regulations everything is tied down in rules and budgets. As such, there is no opportunity to experiment in a certain area or neighbourhood.”* (Participant 1)

One of the research questions was how ‘rebellious’ founders found ways to manoeuvre within the boundaries set by the institutional context and if these founders were able to bend, work around or change certain rules and regulations. First of all, almost all founders emphasised the importance of adhering to the existing laws, rules and regulations, especially as the participants are directors of social housing associations or social entrepreneurs, which means that their aim is not only to make a profit that can be reinvested in the organisation, but also to achieve a societal mission. They found it important to operate in a responsible way:

*“What is very important to me personally, is that we do business in a socially responsible manner. We use public funds and we want to use these funds in the best possible way. If you have other moral standards, I don’t think you are suitable to work in this domain. […] The social responsibility we have is very important to us.”* (Participant 7)

Although the founders mentioned the importance of playing by the rules and the importance to manoeuvre within the existing institutional framework, they also felt a responsibility to challenge and sometimes ignore certain rules, as the rules are not always infallible.

*“We interpret [a certain] law differently so we have had an endless discussion with the Dutch Authority for Social Housing Associations <Dutch: Autoriteit Woningcorporaties>. But in the end, they said: ‘OK, we will tolerate [the difference in interpretation]’. That’s what it comes down to.”* (Participant 2)

The participants were well aware of the opportunities that can be created within the existing legal framework by interpreting the rules and regulations differently:

*“A creative interpretation of the rules is often regarded as something negative. But the legislation offers an opportunity to act and interpret the rules and regulations differently. It is just more like acting in the spirit of the rules than simply [strictly] following the existing rules.”* (Participant 5)

As this quote illustrates, some participants considered it morally legitimate or even morally required to assess existing rules and regulations critically and to challenge them when this seemed justified. For some founders challenging existing rules and acting differently was part of being rebellious. At the same time, they took accountability for their actions and behaviours:

*“I think I am the one within the organisation who encourages people to do that [colour outside the lines] every now and then. Of course, [you do it] in a responsible way: you always need to take accountability for your actions and explain to others what you have done and how you have done it.”* (Participant 13)

Some founders challenged the existing institutional frameworks and also tried to change existing rules or laws, for instance, by lobbying and/or taking action at the local and national levels:

*“In general, people prefer to follow the rules, otherwise there will be a hassle. However, we are prosecuting against the state about the new Housing Act. The government is convinced about a certain interpretation of the Act. We disagree on this, as it is not written down in the Act.”* (Participant 2)

In conclusion, by challenging, bending and sometimes ignoring certain rules or regulations, ‘rebellious’ founders took certain risks. The reputation of a director or social entrepreneur can be put at risk, especially when there is little support inside or outside the organisation for positive deviance. The following quote stems from an interview with a former director, who had been removed from office by resolution of his supervisory board. As a result, the participant was not able to find a new position in the past few years:

*“I am [over 60] years old, but I’m already [living of] my retirement savings. In the first years after I was given the sack, I was still asked to give a presentation or a lecture. But at a certain point of time, people in the field don’t know you anymore and then there are no more assignments. Therefore, I had to apply for pre-pension.”* (Participant 3)

*“So being a rebel does not always pay off?”* (Researcher)

*“No. No. Definitely not.”* (Participant 3)

In order to limit the abovementioned risks, rebellious founders tried to create support both within and outside the organisation. This is further elaborated in the following section.

### 3.3. Creating Supportive Contexts

In order to organise support and legitimate their different way(s) of acting, rebellious founders created supportive contexts, both outside and within their organisation. In order to create a supportive context and to minimise risks of acting differently, it was important for founders to take accountability. Founders took accountability to explain and justify their conduct. First of all, explanations and justifications were made towards the health care inspectorate. This involved providing the inspectorate with information, and, in addition, some founders also tried to discuss their differences in interpretation and how and why they questioned certain rules or regulations. Often, the inspection systems were perceived as rigid. Yet, different founders stated that when communication between founders and inspectors took place, inspection systems proved to be more flexible than often thought:

*“It is assumed that they [the health care inspectorate] need to tick all the boxes. However, if you talk with people from the inspectorate, it becomes slightly different and it appears to be more nuanced.”* (Participant 3)

Founders stressed the importance to formalise the agreements made with the inspectors, especially when certain well-considered risks stemming from acting differently were involved. This applied in particular to founders within the health care sector. They challenged rules and regulations that might impact the quality of life of the residents of the collective housing initiative in a negative manner.

*“We spoke with the inspector before and during the start. […] It is also important for the inspection to discuss the risks with all parties involved and to record what is discussed and include the outcome in the health care plan.”* (Participant 11)

Second, as one founder stated (Participant 11) ‘*being rebellious starts with communication*’. Communication, transparency and accountability were not only of importance towards the inspectorate, but also in creating wider supportive contexts outside the own organisation. This included the broader political context, as the following quote illustrates:

*“Well then we invite a few MPs, or a mayor or a top official. We always invite the new General Secretary: ‘Come and join us for a day.’ And the director of [an inspectorate] did a one-day internship here. Just to understand what and how we are working here.”* (Participant 2)

In creating a wider supportive context founders sometimes used the media and the press strategically:

*“If I am working on something of which I am convinced that it should go in that direction, I use Twitter or LinkedIn to gain support. Often, I receive support from all sides. So, I do approach and use social media to reinforce this.”* (Participant 13)

The illustration given above does not mean founders were always open and transparent and/or used the media to create support. Sometimes, they decided to stay under the radar. Founders strategically chose when, how and with whom they created a supportive context.

Third, some founders took accountability or strategically communicated with external parties, and also tried to create coalitions of like-minded parties and/or people:

*“How do you organise change? I made a list of healthcare organisations, social housing associations and municipalities that are also focused on change. I try to create a coalition of the willing with them.”* (Participant 12)

The purpose of moving from an individual actor to a member of a collective force was to bring people together. By creating a collective force through media attention and/or through coalitions of like-minded people, rebellious founders hoped to be better able to exercise influence and achieve desired and sustainable change.

Yet, founders tried to create supportive contexts both outside the organisation and within. Within the organisation, founders tried to nurture relationships with the supervisory board and employees in order to create supportive contexts. In creating support, several founders tried to keep the supervisory board and their employees inspired and emphasised working towards achieving the same higher goal. One founder (Participant 6) talked about sending their employees to visit an innovative housing facility abroad to inspire them and work accordingly. Founders also looked for allies within the organisation with whom they could share their ideas and thoughts. This worked both ways. One founder stressed the importance for employees to discuss cases and dilemmas in which rules or regulations might be challenged or ignored. Therefore, trust within the organisation and between employees was considered important:

*“Within the organisation employees need to know whether or not they have the support from the board to act differently. You need to feel safe, to know you are supported by the organisation, even if you make a mistake.”* (Participant 5)

As the quote above illustrates, transparency and communication were not only important to create a supportive context externally, but internally, within the organisation, as well.

Second, founders mentioned that a supportive context within the organisation consisted of more than just the employees who are more rebellious:

*“I could always rely on [name of a trusted person]. He kept me with both feet on the ground. Sometimes you might lose sight of reality a little bit. That’s something [this person] was always good at: ‘You may want this, but there are other factors at play here too’.”* (Participant 3)

As the above quotation illustrates, a balance was needed between more rebellious allies or employees and those who could be seen as ‘guardians’, who were able to challenge the founder and discuss potential risks to the organisation.

## 4. Discussion

The study on rebellion in the domain of collective housing for older people revealed three central themes. First, the concept of rebellion was discussed, which, according to the participants, contains four elements: keeping the higher goal in mind; taking action; learning by trial-and-error; and thinking critically instead of blindly following rules. Next, the institutional context was discussed and how responsible rebels sometimes work around, bend or change existing rules and regulations. The final theme concerned the creation of supportive contexts, both within and outside the organisation.

The study contributes to existing literature on ‘positive deviants’, ‘radicals’ and ‘rebels’ in three ways. First of all, while earlier research focused on rebellious teams within ‘traditional’ organisations [14], this study was about founders of rebellious initiatives, who not only have to create supportive contexts within their organisation, but also have to create coalitions of the willing with external stakeholders.

Second, the founders operate on the intersection of two domains, namely housing on the one hand and care on the other. In order to be successful, they have to know the rules of the game of both worlds. As van Straaten et al. [13] showed, experienced founders know how to communicate with municipalities, which are an important player in the Netherlands in both domains. This study adds that founders use various strategies to legitimise their initiative to different stakeholders. Based on their trial-and-error learning style—which they described as central to the innovative character of their initiatives—they know what works under what circumstances. For instance, which rules should be adhered to and which ones can be circumvented or ignored, or when to seek publicity and when staying under the radar seems to be more effective.

Third, the study showed that the terms ‘rebel’ and ‘rebellious’ are embraced by some but not all founders of innovative living arrangements. Earlier studies used terms such as ‘rebels’ and ‘radicals’ without questioning whether the participants actually identified themselves with these terms. This study shows that the ‘rebellious’ founders studied had different opinions on the term. Some were very positive about it and even said that the use of the term in the project title was the reason for them to participate in the research. Some others did not have strong positive or negative feelings about it. A third group did not identify with the term, because for them, it had a negative connotation, for instance, because the term suggested that they were fighting others, while in reality, they tried to create alliances.

Based on the above-described contributions, the findings of this study suggest that the rebellious founders are not just rebellious, but also responsible. They find it important to challenge existing formal and informal rules, both within and outside their organisations, but they want to do this in a responsible manner. We coin this ‘responsible rebellion’, which means that the founders stress the importance of playing by the rules and to manoeuvre within the existing institutional framework on the one hand, but on the other hand also challenge and sometimes ignore certain rules. Founders communicate with and take accountability for explaining and justifying their conduct to external and internal stakeholders. As such, being a ‘responsible rebel’ can lead to innovative forms of group housing for older people, which less ‘rebellious’ organisations fail to deliver. Additionally, although they take some risks, the participants are all highly committed to their organisations. Being a responsible rebel implies ‘walking the tightrope’, as these rebels ‘make sure not to harm their organisation and they nurture relationships’ but at the same time they sometimes disobey certain rules ‘to stay close to their ideas and convictions’ [14], p. 879.

There are clear connections between the results of the present study, and previous findings on how to create so-called age-friendly cities. If you consider the foundation of new housing initiatives from the perspective of age-friendly cities and the domain of housing, one needs to look at key characteristics of such age-friendly cities which were reviewed by Steels [25]. Steels found a fruitful collaboration between the different stakeholders, including local and national governments concerning financial and political support, and the involvement and social inclusion of older people themselves, to be important factors. These findings overlap with the perceived barriers of the research participants, who often deal with the boundaries set by the government, a good collaboration with various stakeholders, and the facilitation of participation and having a say of older people.

In addition, the development, design and construction of a new collective housing initiative is a process that can last for many years, and which follows a long administrative process and private and public consultation with a large number of parties. Such initiatives have a mixed level of success [26]. In an administrative landscape, which is the result of previous excesses in the domain of public housing, social entrepreneurs often refer to public administrations as organisations that pose barriers to their initiatives. Internal regulations, the required collaboration and interaction with other stakeholders, can contribute to further delays and barriers for social entrepreneurs when they engage in the development of housing initiatives for older people. Social entrepreneurs want to enjoy a degree of freedom to develop a new concept and bring its services to the marketplace, but supervisory board members are often critical about the quality, durability and sustainability of such initiatives.

Therefore, to set up innovative housing for older people, responsible rebels are needed. For future research, the role of supervisory boards and external inspectorates in how they deal with responsible rebellion among directors within their organisation needs attention. What supervisory styles do they use and when? Do they hinder or stimulate responsible rebellion within their organisation and why? And how do they want their directors to be accountable? Lessons might be learned from the health care sector in which a programme was designed to find new ways of accountability. This ‘narrative accountability’ is based on a more ‘story conscious’ way of engaging with the realities of life and care [27]. Moreover, a critical assessment is needed of what the added value of rebellion is for the quality of housing and care. What do rebellious initiatives yield in output compared to ‘traditional’ initiatives? Are they able to fulfill their ambitions [13]? Furthermore, future research should focus on the participation of older people in decision making concerning new housing concepts. In this study, the focus was on the importance of creating supportive contexts, by nurturing relationships internally, as well as externally [14]. Yet, how are the residents of the collective housing initiatives involved? Are the ‘rebellious’ initiatives age friendly according to the (future) residents [28,29]? Does the housing initiative contribute to the quality of life of its residents and does the housing initiative fulfil their needs and wishes?

Another important aspect that is of interest for further study is the role financing plays in relationship to the level of rebelliousness. For instance, social housing associations in the Netherlands are bound to an extensive set of financial schemes and regulations, which help protect the financial integrity of the system. Social entrepreneurs who are not bound by these rules have more freedom in seeking and securing a loan from the capital market. The degree of having to adhere to a prescribed system of checks and balances as a founder may impact the level of freedom that founders perceive in their operation. Moreover, housing has a maximum asking price in terms of rent, if a tenant was to stay eligible for rental allowance from the state. Higher rents can be asked but will attract a different group of more affluent tenants. These tenants often own a home and are less prone to sell their house and start renting. Such financial contexts are specific to the Netherlands and impact the applicability of the current findings in an international context. Each country in the European Union has its own cultural and governance contexts when it comes to housing and care for older people, as these two domains are still the responsibility of national governments.

## 5. Conclusions

The establishment of collective forms of housing for older people in the Netherlands goes together with social entrepreneurship that—when successful—often deviates from the mainstream housing arrangements. The ways that founders dealt with challenges in establishing and governing such initiatives were studied in relation to the concept of ‘rebellion’. Rebellion, in the words of the participants, revolves around four elements: keeping the higher goal in mind; taking action; learning by trial-and-error; and thinking critically instead of blindly following rules. The in-depth interviews with founders showed that they encountered various obstacles. Such obstacles are often related to governmental and sectoral rules and regulations, which are specific for each of the domains of housing and care, and which may intersect in the domain of housing for older people. Knowing how to work around them and deal with existing rules and regulations is critical to being successful in the field. In order to succeed in realising plans, one needs to create supportive contexts, both within their own organisations and—no less important—with external stakeholders. Being a ‘responsible rebel’ can indeed lead to innovative forms of group housing for older people, which less ‘rebellious’ organisations fail to deliver. Being a ‘responsible rebel’ is, however, not entirely without risks. If the behaviours displayed and choices made are too challenging, and boundaries are crossed on a continuous basis, irresponsible behaviours may bring a project (or even a career) to a halt. First and foremost, being a responsible rebel is about knowing how to walk the tightrope within the context of the country in which one works.

## Figures and Tables

**Table 1:** Overview of the participants.

Participant	Age-Range [Years]	Sex	Position
01	40–49	female	social entrepreneur
02	50–59	male	director
03	≥60	male	director
04	30–39	male	director
05	50–59	female	director
06	50–59	male	director
07	50–59	male	director
08	50–59	male	director
09	≥60	male	member supervisory board
10	≥60	male	social entrepreneur
11	≥60	male	social entrepreneur
12	≥60	male	director
13	50–59	female	member supervisory board
14	40–49	female	director
15	≥60	male	social entrepreneur
16	≥60	male	social entrepreneur
17	50–59	male	social entrepreneur

## Supplementary Materials

The following are available online at https://www.mdpi.com/1660-4601/17/17/6235/s1.
