# Supplementary material for: Towards Responsible Rebellion: How Founders Deal with Challenges in Establishing and Governing Innovative Living Arrangements for Older People
Source: Int J Environ Res Public Health. 2020 Aug 27;17(17):6235. doi: 10.3390/ijerph17176235 (PMC7503804; doi:10.3390/ijerph17176235)
Supplement: Supplementary file 1 [file ijerph-17-06235-s001.pdf]

International Journal of
Environmental Research and Public Health

MDPI

Supplementary Materials: Towards Responsible Rebellion: How Founders Deal with Challenges in Establishing and Governing Innovative Living Arrangements for Older People

File 1. Topic List Social Entrepreneurs

Explanation of the research

- A study about new collective housing initiatives for and by older people.
- Rebellious initiatives, although the definition and boundaries of 'rebellion' are still undefined.
- We were referred to you because you have an initiative that is 'outside of the box'. How do you see this?

Initiative

- Can you tell us a bit more about your housing initiative?
    - What problem does this housing initiative address?
    - Has this initiative actually resolved the problem?
    - How location and time specific is this initiative? Can it also be applied elsewhere?
    - How is the target group itself involved in the implementation of this initiative?
    - How is the target group involved in the day-to-day activities? Or: how much of a voice do they have? Or: in which way do they influence the administration of the initiative?
    - Going one step further: how is the target group encouraged to take ownership of their own life and how is this made possible and ensured within the organisational side of the initiative?
- When did you as an entrepreneur start the initiative? When did the housing initiative actually start?
    - Why did you start this initiative? Personal motivation? What motivates you?
    - What is your vision on housing/care?
    - Are you a frontrunner/pioneer with this initiative? In what way?

Previous experience

- What did you do before you became an entrepreneur?
- Did you gain any knowledge and experience in your (previous) work that is relevant for this current initiative? What does this consist of?

The term rebellion

- As mentioned above, we are studying rebellious housing initiatives. How do you define rebellion? What does this term evoke for you (either positive or negative associations)?
    - When positive/when negative?
    - When does rebellion become a negative label?
    - Where do you draw the line for yourself?
- Do you see yourself as a rebel? What character traits define you?
- Who is an example of a rebel to you? Is this person an inspiring example (or does this person cross the line)? And why?
    - How does this person differ from you?

- What are the similarities?
- What do you think when entrepreneurs, directors and/or members of the supervisory board take some creative license with rules or policies to do good, or put other issues on the agenda than the systems require and try to go their own way?
- What entrepreneurs, directors and/or members of the supervisory board would you describe as rebellious and why?

Good care/good housing

- How do you define quality housing and quality care? How do you try to achieve this in your initiative?
- Do you sometimes break the rules? When and when not? Do you know how far you can go and when you have gone too far? What is the personal cost to you?
- And to whom or what are you accountable?
- What do you need to do/create within or outside the context of the organisation to be successful/deliver good care?

Dealing with legislation, regulations, licenses and organisations

- What legislation and regulations, licenses, authorities and organisations did you have to deal with in setting up your housing initiative? Is there something that stood out for you?
  - What do you think of the quality system/indicators/protocols around care and housing?
- Do these hinder or help?
- Have you ever gone against legislation/regulations? If yes, what legislation/regulations and with whom/what?
  - Did you feel restricted by members of the supervisory board or authorities in that case? If yes, how and by whom?
  - To what extent do you take a personal/business risk? Where do you draw the line?
- Is there any leeway in complying with legislation/regulations? Where do you find this leeway?
- Do you try to influence/change any regulations, and if yes, how?
- Do you feel that in recent years flexibility in dealing with legislation and regulations (for example with external supervisors) has increased or decreased?

Partners & network

- Who were the most important (in)formal partners in setting up this housing initiative? And in what ways?
- What do you need from different partners to develop a successful initiative?
  - What do you need from partners, participants and the target group, and what do you need from the staff members of the initiative itself to continue and ensure a successful initiative?
- Are there 'rebels' among your partners? Who are they? And in what way are they rebels? Do they work in the same sector?
- What do you look for when you choose partners or staff members (do you look for rebels or not at all)?
- Do you share experiences/knowledge with your network/partners?
  - How do you share knowledge? Internet/association/networking
  - In what way (and in what media) do you communicate about the initiative?
  - Are successes and milestones celebrated and shared? With whom?
- Are there parties that you avoid professionally and why?
- Who are your competitors and in what way?

Funding/financing

- Did you make a business plan beforehand and was it feasible?
- How did you arrange the funding/financing? Does the initiative fit entirely within officially available funding/financing structures or were any additional resources required?
    - If yes, what additional resources did you seek out and were you able to obtain these?

Risk management

- Did you perform a risk assessment before starting this initiative?
    - If yes, what were the biggest risks and how were those managed?
    - Think of: real estate, project development, information (reliable data) staff, fiscal issues, safety of residents.
- What risks are you absolutely not willing to take? Do you know any entrepreneurs who would take these risks?

Accountability

- Does the initiative have a board and who are the internal (members of the supervisory board) and, possibly, external supervisors?
    - What is the relationship like with the (supervisory) board members?
- To whom are you (in terms of finances and operations) accountable?
- How do you provide accountability (including any social media)?
- Are you satisfied with the position of the members of the supervisory board and external supervisors? Are there things that you feel they should do differently/better?
- How do you want staff members to be accountable? Are there (rebellious) things they do that you don't know about or don't want to know about?
- How are the residents engaged in the initiative?
    - How is participation arranged?
    - Does this play an important role in the initiative, or not?
    - What works well and what doesn't work well when it comes to resident participation?
- How do you promote the housing initiative to the broader public (for example, via social media)?

Future

- What are your plans for the future? For example, scaling up the initiative, new initiatives?
- What role do you see here for yourself? As a rebel or not?
- Where do you hope that you/the initiative will be in five years?

Background information respondent

- Age:
- Highest completed degree:

Conclusion

- Who else should we involve in our research? What rebellious entrepreneur, director and/or member of the supervisory board should we speak with and why?

File 2. Topic List Directors

Explanation of the research

- A study about new collective housing initiatives for and by older people.
- Rebellious initiatives, although the definition and boundaries of 'rebellion' are still undefined.
- We were referred to you because as director you are involved in a housing initiative where things are done somewhat differently, or because you are known as a rebellious director. How do you see this?

Initiative

- ***Interviewer describes an example of a collective housing initiative where things are done somewhat differently, in which the respondent was involved.***
- Can you tell me more about this **housing initiative** (when did it start, etc.)?
- Who was the **initiator**?
- **What problem** does this housing initiative address?
  - Has this initiative actually resolved the problem? If not, what would it require?
- How **location and time specific** is this initiative?
- How is the **target group itself involved** in the implementation of this initiative?
  - Participation: Inform, have a say, consult, co-produce, delegate (hand over tasks), self-administration
  - How is the target group involved in the day-to-day activities? Or: how much of a **voice** do they have? Or: in what way do they influence the director(s) of the initiative?
  - Going one step further: how is the target group encouraged to **take ownership of their own life** and how is this made possible and ensured within the organisational side of the initiative?
- Why is this initiative important to you?
  - **Personal motivation?** What motivates you?
- Are you a **frontrunner/pioneer** with this initiative in the sector? In what way?
- Does the initiative have a **board** and who are the internal and, possibly, external **supervisors/members of the supervisory board**?
  - How is the **relationship** with the members of the supervisory board involved and the entrepreneur?
- How did you arrange **funding/financing**?
  - Did the initiative fit entirely within officially available **funding/financing structures** or were any additional resources required?
    - If yes, what **additional resources** were pursued and were these obtained?
  - What **parties** are involved in funding/financing?
- How do you promote the housing initiative to the **broader public** (for example, via social media)?

Risk management

- Did you perform a **risk assessment** before starting this initiative?
  - If yes, what were the **biggest risks** and how were those managed?
  - Think of: real estate, project development, information (reliable data) staff, fiscal issues, safety of residents.
- What risks are you **absolutely not willing to take** as director? Do you know any directors who would?
- What are the most important factors in the success or failure of the initiative?

The term rebellion

- As mentioned before, we are analysing rebellious housing initiatives.
  - Why is the example above **an example (or not) of a rebellious initiative**?
- How do you define **rebellion**? What does this term evoke for you (either positive or negative associations)?
  - When **positive**/when **negative**?
  - When does rebellion become a negative label?
  - Where do you draw the **line**?
- Do you see **yourself as a rebel**? What character traits define you?
- Who is an **example of a rebel to you (entrepreneur/director/member of the supervisory board)**? Is this person an inspiring example (or does this person cross the line)? And why?
  - How does this person differ from you?
  - What are the similarities?
- What do you think when entrepreneurs, directors and/or members of the supervisory board take some **creative license with rules or policies** to do good, or put other issues on the agenda than the systems require and try to go their own way?
  - Do you take creative license with the rules or policies? If yes, how?

Good care/good housing

- How do you define **quality housing** and **quality care**? How do you try to achieve this as a director?
- Do you **test the limits**? If yes, **how**? When would you do this and when would you not? Do you know how far you can go and when you have gone too far? What is the personal cost to you?
- And to whom or what are you **accountable** about this?
- What do you need to do/create within or outside the context of the organisation **to be successful**/deliver good housing quality and quality care?
- What do you need from **different partners** to develop a successful housing initiative?

Dealing with legislation, regulations, licenses and organisations

- What **legislation and regulations, licenses, authorities and organisations** do you have to deal with as a director?
  - What do you think of the **quality system/indicators/protocols** around care and housing?
  - Do these **hinder** or **help**?
- Have you ever gone **against legislation/regulations**? If yes, what legislation/regulations and with whom/what?
  - Do you feel **restricted (or not)** by members of the supervisory board, authorities and others? If yes, how and by whom?
  - To what extent do you take a **personal/business risk**? Where do you draw the line?
- Is there any **leeway** in complying with legislation/regulations? Where do you find this leeway?
- Do you try to **influence/change any regulations,** and if yes, how?
- Do you feel that in recent years **flexibility** in dealing with legislation and regulations (for example with external supervisors) has **increased or decreased**?

Partners & network

- Who are the most important **(in)formal partners** for you in your role as director? And in what ways?
- Are there **'rebels' among your partners**? Who are they? And in what way are they rebels? Do they work in the same sector?

- What do you look for when you **choose partners** or staff members (do you look for rebels or not at all)?
- Do you **share experiences**/knowledge with your network/partners?
    - How do you share knowledge? Internet/association/networking
    - In what way (and in what media) do you communicate about the initiative?
    - Are successes and milestones celebrated and shared? With whom?
- Are there parties that you **avoid** professionally and why?
- Who are your **competitor**s and in what way?

<u>**Accountability**</u>

- **To whom** are you (in terms of finances and operations) accountable?
- **How** do you provide accountability (including any social media)?
- Are you **satisfied** with the position of the internal members of the supervisory board and external supervisors? Are there things that you feel they should do differently/better?
- How do you want **staff members** to be accountable?
    - Are there (rebellious) things they do that you don't know about or don't want to know about?
- What do you as a director **need from a supervisory board**?
    - As a **rebellious** director do you need to provide **extra accountability** to the supervisory board?

<u>**Future**</u>

- What are your **plans for the future**? For example, scaling up the initiative, new initiatives?
- What **role** do you see here **for yourself**? As a rebel or not?
- Where do you hope that you/the initiative will be i**n five years**?

<u>**Background information respondent**</u>

- Where did you work before you became a director?
- Were you known in your previous job as someone who did things differently? If yes, in what way?
- Age:
- Highest completed degree:

<u>**Conclusion**</u>

- Who else should we involve in our research? What rebellious entrepreneur, director or member of the supervisory board should we speak with and why?

File 3. Topic List Members of the Supervisory Board

Explanation of the research

- A study about new collective housing initiatives for and by older people.
- Rebellious initiatives, although the definition and boundaries of 'rebellion' are still undefined.
- We were referred to you because as a member of the supervisory board you are involved in a corporation where things are done somewhat differently, or because you are known as a rebellious supervisor. How do you see this?

<u>Background respondent</u>

- Since when have you been an supervisor? For what organisation(s)?
    - Other duties in addition to your role as supervisor?
- Can you tell us more about the organisation(s) where you are active as supervisor?
- What are your most important duties as an supervisor?
- Why did you become an supervisor?
    - **Personal motivation?** What motivates you?

<u>Initiative</u>

- Can you give an example of a housing (care) initiative where things were done/are done differently and in which you were/are involved as supervisor?
    - Can you tell more about this **housing initiative** (when did it start, etc.)?
    - Who was the **initiator**?
    - **What problem** does this housing initiative address?
        - Has this initiative actually resolved the problem?
- Was there a lot of discussion within the Supervisory Board about this initiative? Or was there broad support for it?
- What was your role in this initiative?
    - Only as an supervisor? If yes, what did you mainly oversee?
    - Stimulating/supportive/enthusiastic/restraining attitude towards the board?
- Why is this initiative important?

- Was a **risk assessment** performed before starting this initiative?
    - If yes, what were the **biggest risks** and how were those managed?
    - Think of: real estate, project development, information (reliable data) staff, fiscal issues, safety of residents.

- Does the initiative fit entirely within officially available funding/financing structures or were any additional resources required?
    - If yes, what **additional resources** were pursued and were these obtained?
    - What **parties** are involved in funding/financing?

- How is the **target group itself involved** in the implementation of this initiative?
    - How is the target group involved in the day-to-day activities? Or: how much of a **voice** do they have? Or: in which way do they influence the administration of the initiative?
    - Going one step further: how is the target group encouraged to **take ownership of their own life** and how is this made possible and ensured within the organisational side of the initiative?

- How is the housing initiative promoted to the **broader public** (for example via social media)?

Supervision (styles)

- What are the **most important challenges** for an supervisor?
- What are the **most important conditions** for supervision to work?
- How do you stay **up to date on (relevant) developments** in the sector?
- How do you determine if you have adequately **mapped out the risks**?
- How would you **describe your own supervision style**?
    - Please note: Focus on the learning capacity of the organisation, 'trust unless', enforcement, controlling
- How would you describe the **supervision style of your colleagues**?
- Have you ever had to **intervene as supervisor**? If yes, when and why?
- How is the **relationship with the director**(s)?
    - Freedom versus control
- What **risks are you absolutely not willing to take** as supervisor? Do you know supervisors who would?

The term rebellion

- How do you define **rebellion**? What does this term evoke for you (either positive or negative associations)?
    - When **positive**/when **negative**?
    - When does rebellion become a negative label?
    - Where do you draw the **line**?
- Do you see **yourself as a rebel**? What character traits define you?
    - Are you known in your job/role as supervisor as someone who does things differently? If yes, in what way?
- Can **rebellion and supervision go hand in hand**?
- Who is an **example of a rebel to you (entrepreneur/director/supervisor)**? Is this person an inspiring example (or does this person cross the line)? And why?
    - How does this person differ from you?
    - What are the similarities?
- What do you think when entrepreneurs, directors and/or supervisors take some **creative license with rules or policies** to do good, or put other issues on the agenda than the systems require and try to go their own way?

Good care/good housing

- How do you define **quality housing** and **quality care**? How do you try to achieve this as supervisor?
- Do you **test the limits**? When would you do this and when would you not? Do you know how far you can go and when you have gone too far? What is the personal cost to you?
- And to whom or what are you **accountable** about this?

Dealing with legislation, regulations, licenses and organisations

- What **legislation and regulations, licenses, authorities and organisations** do you have to deal with as an supervisor?
    - What do you think of the **quality system/indicators/protocols** around care and housing?
    - Do these **hinder** or **help**?
- Have you ever gone **against legislation/regulations**? If yes, what legislation/regulations and with whom/what?
    - Do you feel **restricted (or not)** by supervisors, authorities and others? If yes, how and by whom?

    - To what extent do you take a **personal/business risk**? Where do you draw the line?
- Is there any **leeway** in complying with legislation/regulations? Where do you find this leeway?
- Do you try to **influence/change any regulations,** and if yes, how?
- Do you feel that in recent years **flexibility** in dealing with legislation and regulations (for example with external supervisors) has **increased or decreased**?

<u>Partners & network</u>

- Who are the most important **(in)formal partners** for you in your role as supervisor? And in what ways?
- Are there **'rebels' among your partners**? Who are they? And in what way are they rebels? Do they work in the same sector?
- Do you **share experiences**/knowledge with your network/partners?
    - How do you share knowledge? Internet/association/networking
    - In what way (and in what media) do you communicate about the initiative?
    - Are successes and milestones celebrated and shared? With whom?

<u>Accountability</u>

- Are you **satisfied** with the position of the internal and external supervisors? Are there things that you feel they should do differently/better?
- How do you want **directors** to be accountable?
    - Are there (rebellious) things they do that you don't know about or don't want to know about?
- What do you as supervisor **need from a director to provide good supervision**?
    - Should a **rebellious director or supervisor be even more accountable**?

<u>Future</u>

- What are your **plans for the future**?
- What **role** do you see here **for yourself**? As a rebel or not?
- Where do you hope that you/the initiative will be i**n five years**?

<u>Background information respondent</u>

- Age:
- Highest completed degree:

<u>Conclusion</u>

- Who else should we involve in our research? What rebellious entrepreneur, director or supervisor should we speak with and why?
